# Insular cortex corticotropin-releasing factor integrates stress signaling with social affective behavior

**DOI:** 10.1038/s41386-022-01292-7

**Published:** 2022-02-26

**Authors:** Nathaniel S. Rieger, Juan A. Varela, Alexandra J. Ng, Lauren Granata, Anthony Djerdjaj, Heather C. Brenhouse, John P. Christianson

**Affiliations:** 1 Department of Psychology & Neuroscience, Boston College, 140 Commonwealth Ave, Chestnut Hill, MA 02467 USA; 2 Psychology Department, Northeastern University, 360 Huntington Avenue, 115 Richards Hall, Boston, MA 02115 USA

**Keywords:** Stress and resilience, Social neuroscience, Insula

## Abstract

Impairments in identifying and responding to the emotions of others manifest in a variety of psychopathologies. Therefore, elaborating the neurobiological mechanisms that underpin social responses to social emotions, or social affective behavior, is a translationally important goal. The insular cortex is consistently implicated in stress-related social and anxiety disorders, which are associated with diminished ability to make and use inferences about the emotions of others to guide behavior. We investigated how corticotropin-releasing factor (CRF), a neuromodulator evoked upon exposure to stressed conspecifics, influenced the insula. We hypothesized that social affective behavior requires CRF signaling in the insular cortex in order to detect stress in social interactions. In acute slices from male and female rats, CRF depolarized insular pyramidal neurons. In males, but not females, CRF suppressed presynaptic GABAergic inhibition leading to greater excitatory synaptic efficacy in a CRF receptor 1 (CRF_1_)- and cannabinoid receptor 1 (CB_1_)-dependent fashion. In males only, insular CRF increased social investigation, and CRF_1_ and CB_1_ antagonists interfered with social interactions with stressed conspecifics. To investigate the molecular and cellular basis for the effect of CRF we examined insular CRF_1_ and CB_1_ mRNAs and found greater total insula CRF_1_ mRNA in females but greater CRF_1_ and CB_1_ mRNA colocalization in male insular cortex glutamatergic neurons that suggest complex, sex-specific organization of CRF and endocannabinoid systems. Together these results reveal a new mechanism by which stress and affect contribute to social affective behavior.

## Introduction

Stressors and other salient emotional stimuli trigger a shift in attention and cognitive resources in order to orient attention and organize situationally adaptive behaviors. In the brain, the transition between resting and executive cognitive networks involves the insular cortex and the “salience network” [1, 2], which is anatomically situated to integrate sensory, emotional and cognitive processes [3, 4]. Not surprisingly, insula is associated with many cognitive functions and, in human neuroimaging studies, insula activity correlates with emotion recognition, pain, drug craving and anticipatory fear [5–8]. Specifically, the posterior insular cortex in rodents integrates external sensory cues with internal states and is interconnected with a number of subcortical regions to modulate behavior in response to these cues in a top-down fashion [4, 9, 10]. Aberrant activity and functional connectivity of the posterior insula and associated network structures leads to hypervigilance, increased interoception and poor emotion regulation—hallmark symptoms of many neuropsychiatric disorders including autism spectrum disorders, schizophrenia, and posttraumatic stress disorder [11].

Stress is a major precipitating factor for mental illness. Exposure to either a perceived threat to one’s own well-being (self stress), or to a social contact that is undergoing distress initiate the hypothalamic–pituitary–adrenocortical axis response by activation of corticotropin-releasing factor (CRF) neurons in the paraventricular hypothalamus (PVN) [12]. The CRF system is complex, consisting of 2 receptor subtypes (CRF_1_ and CRF_2_) that couple to a variety of G-proteins expressed in the brain region, cell type, and sex-specific ways [13, 14]. CRF_1_ and CRF_2_ receptors are distributed throughout brain social behavioral networks making CRF an important contributor to many social behaviors [15]. CRF_1_ receptors and CRF immunoreactive fibers are expressed throughout the corticolimbic system, including in the insular cortex [16–19]. Observing others in distress is highly salient and a potent driver of insular activity that is thought to contribute to empathic cognition [20, 21]. A fundamental precursor to empathy is emotion contagion, a primitive process by which the affective state of a demonstrator leads to a complementary state in the observer [22]. CRF neurons of the PVN are activated upon exposure to stressed conspecifics that is a mechanism providing for the social transfer of stress responses [12]. CRF may shape social behaviors by actions at CRF receptors located among the distributed network of neural structures, including the insular cortex, that are engaged by social stress signals and organize social behavior [23].

Seeing CRF as a putative modulator of insula and considering the significant influence of stress on psychosocial processes, we investigated the effects of CRF on insular physiology and social affective behavior. In whole-cell recordings of insular pyramidal neurons, CRF depolarized the membrane potential. This translated to an increase of excitatory synaptic transmission, but only in recordings from male rats. The gain of synaptic efficacy appeared to be a case of CRF causing depolarization-induced suppression of inhibition (DSI [24]) as the effects of CRF were dependent on both GABA_A_ and cannabinoid type 1 receptor (CB_1_). In social behavior, CRF augmented social investigation while a CRF_1_ antagonist interfered with social interactions with stressed conspecifics in male but not female rats. Because we observed sex-specific effects of CRF on physiology and behavior, we hypothesized that sex differences exist in CRF_1_ and CB_1_ at the cellular and molecular levels. We employed a combination of quantitative polymerase chain reaction (qPCR) and anatomical (in situ hybridization) analyses and found sex differences in CRF_1_ mRNA expression and differences in the cellular distribution of the transcripts in the insular cortex. Together, the data lead us to conclude that CRF, acting upon CRF_1_ receptors depolarizes pyramidal neurons triggering the release of endocannabinoids which suppress presynaptic inhibition. The result facilitated flow of information through the insula that appears to be necessary for coordinating social interactions with stressed conspecifics.

## Materials and methods

For a complete description of the experimental methods please refer to the Supplementary Materials.

### Animals

Male and Female Sprague-Dawley rats were obtained from Charles River Laboratories (Wilmington, MA) at either age PN45 (test rats and adult conspecifics) or PN21 (juvenile conspecifics) and maintained in the same-sex groups of two to three. All procedures were conducted in accordance with the NIH Guide for the care and use of laboratory animals and approved by the Boston College Institutional Animal Care and Use Committee.

### Electrophysiology

Whole-cell and extracellular field potential recordings were obtained from acute insular cortex slices from adult male and female rats with methods reported previously [21]. Active and passive intrinsic membrane properties were determined with current clamp recordings with the glutamatergic receptor blocker kynurenic acid (1 mM) and the GABA_A_ receptor antagonist SR95531 (2 µM) in the recording aCSF to block synaptic transmission. After achieving a whole-cell configuration, baseline recordings were made in aCSF until 10 min of stable baseline were observed, at which point 50 nM CRF (human/rat, Cat. No 1151, Tocris) was added to the bath. The dose of 50 nM was selected after a pilot study using a range of doses from 50 to 300 nM, representative of the low [25] and high [26] doses found in the literature. While dose responses were evident in field recordings, no dose responses were seen in intrinsic measures between 50 and 300 nM concentrations. Therefore, patch-clamp experiments utilized 50 nM CRF.

Evoked field excitatory postsynaptic potentials (fEPSPs) were recorded on a perforated multielectrode array. CRF (50 or 300 nM), CRF_1_ antagonist CP154526 (10 µM, the dose that prevented CRF effects on intrinsic properties and comparable to other studies [26]), and GABA_A_ inhibitor SR95531 (2 µM, a dose that eliminates spontaneous and evoked IPSCs in our preparation, Varela JA and Christianson JP, unpublished data) were dissolved in water or DMSO and then diluted to their final concentration in aCSF and bath applied. Input/output curves were established at baseline (aCSF) and again after 10 min of drug application. Stimulations ranged from 0 to 5 V and occurred in biphasic (220 µs) 500 mV increments.

To determine the effect of CRF on GABA_A_-mediated evoked inhibitory postsynaptic currents (eIPSCs), synaptic responses were evoked by a bipolar extracellular stimulating electrode in the presence of glutamatergic antagonists during voltage-clamp recordings of insular pyramidal neurons. eIPSCs were quantified as the peak amplitude observed in the 1 ms post stimulation and converted to z-scores using the mean and standard deviation of the baseline (aCSF).

### Surgical implantation of insula cannula and microinjection

Bilateral guide cannula was implanted in the posterior insular cortex. The posterior insula was targeted because it is functionally connected to the social decision-making network [10, 27], necessary to social affective processing [28, 29] and contains mRNA for CRF_1_ receptor [17, 30, 31]. For insula microinjections, CRF was dissolved in DI water and diluted to 300 nM concentration in a vehicle of 0.9% saline. CRF_1_ receptor antagonist CP154526 (Sigma) and CB_1_ receptor inverse agonist AM251 (Tocris) were dissolved in DMSO then diluted to 10 or 2 µM, respectively, in a vehicle of 10% DMSO and 0.9% saline.

### Social exploration

One-on-one social interaction tests were completed in a quiet room as previously described [29, 32]. The test began with the introduction of either a juvenile (28 ± 2 days old) or adult (50 ± 2 days old) same-sex conspecific. Rats were then allowed to interact for 5 min and interactions were scored for social behaviors (sniffing, pinning and allogrooming) initiated by the test rat by an observer blind to treatment. Rats were tested on consecutive days. For CRF testing, rats received either vehicle or CRF in a counterbalanced order. For CRF_1_ antagonist and AM251 experiments, rats received 4 treatments (vehicle, CRF, CRF_1_ antagonist, or CRF + CRF_1_ antagonist) or (vehicle, CRF, AM251, CRF + AM251), respectively, on consecutive days with treatment order counterbalanced in a Latin square design.

### Social affective preference (SAP) test

The SAP tests allow for the quantification of social interactions initiated by a test rat toward either a stressed or unstressed conspecific, providing insight into the test animal’s discrimination of socioemotional affective cues; they were conducted exactly as previously described [29, 32, 33]. Briefly, the SAP test begins when a test rat is placed in the center of an arena containing chambers on opposite sides containing conspecifics. The test rat was allowed to interact with the conspecifics for 5 min and time spent body sniffing and reaching for the conspecific was recorded. Microinjections were made 30–40 min prior to tests in which one of the conspecifics placed into the cage was stressed via two, 5 s 1 mA footshocks (60 s interval) immediately before testing and the other conspecific was naive to stress. Microninjections consisted of either vehicle, CRF_1_ antagonist or CB_1_ antagonist as described above in a counterbalanced, within-subject design.

### Insular mRNA quantification

To determine the relative expression of CRF_1_ (*crhr1*), CB_1_ (*cnr1*) CRF (*crh*), and CRF_2_ (*crhr2*) mRNA we performed quantitative Taq-man reverse transcriptase qPCR analysis on 1 mm dia, ~500-μm thick insular cortex punches, as previously described [34].

### RNAScope in situ fluorescent hybridization

RNAScope was performed on insular cortex sections according to the vendor’s instructions (ACDBio). Briefly, tissue was thawed, fixed and treated with a RNAScope cocktail including probes for CRF_1_ (*crhr1*, catalog #318911), CB_1_ (cb1, catalog #412501), vesicular glutamate transporter 1 (*vglut1, catalog #317001*) and DAPI. The total number of nuclei (DAPI) and glutamate cells (Vglut+DAPI), and cells colocalized with CRF_1_, CB_1_ or both were determined. Effects of sex on cell counts were assessed in two-way ANOVAs with sex as a between-group factor and side as a within-subject factor. The mean of left and right hemisphere counts are shown.

### Statistics

All analyses were performed in Prism (GraphPad, version 9.0.2) and results of analysis of variance, *t*-tests, final sample size and post hoc test results are presented in the figure legends.

## Results

### Corticotropin-releasing factor depolarizes insular cortex pyramidal neurons

**Fig. 1:**
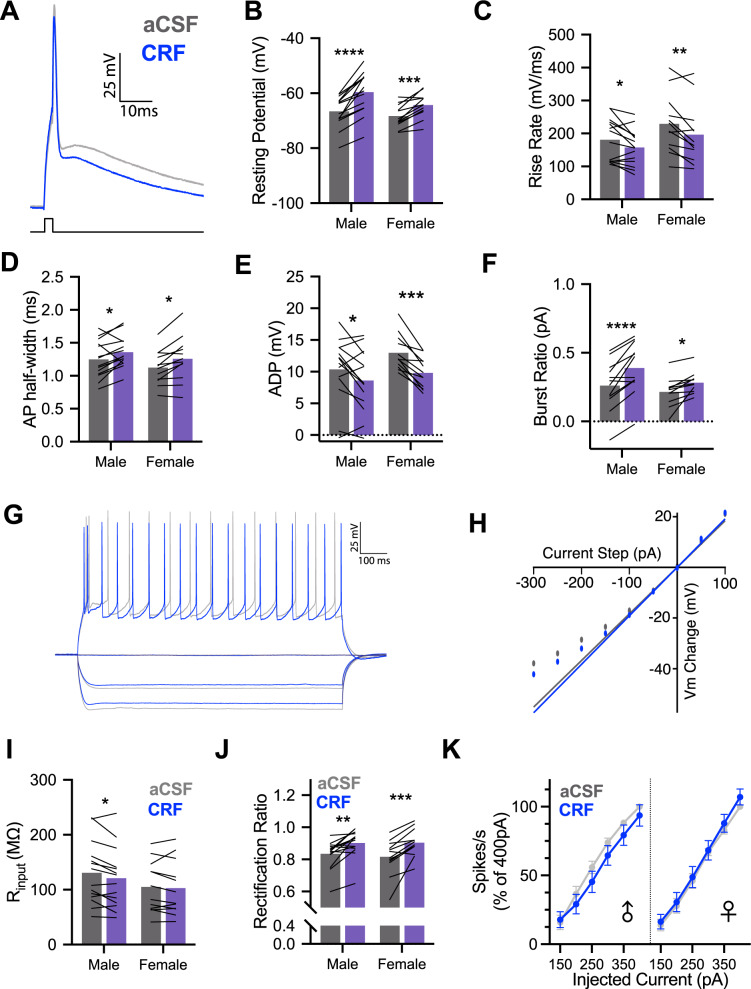
CRF alters intrinsic properties of male and female insular cortex pyramidal neurons in whole-cell recordings. **A** Representative single action potential (AP) recordings of deep layer insular cortex pyramidal neurons at baseline (aCSF-gray) and after application of 50 nM Corticotropin-releasing factor (CRF-blue). **B** CRF decreased the resting potential of male (*n* = 14) and female (*n* = 12) pyramidal neurons, *F*_CRF_(1, 24) = 72.93, *P* < 0.0001, with this effect being stronger in males than females as indicated by a CRF × sex interaction, *F*_CRF × SEX_(1, 24) = 5.124, *P* = 0.033. **C** Action potential rise rate was reduced by CRF in both males and females, *F*_CRF_(1, 24) = 18.93, *P* = 0.0002. **D** Action potential half-width increased following CRF application in male and female recordings, *F*_CRF_(1, 24) = 16.69, *P* = 0.0004. **E** CRF reduced the amplitude of the after depolarization (ADP) in both male and female recordings, *F*_CRF_(1, 24) = 26.83, *P* < 0.0001. **F** CRF increased the current required to trigger burst firing in male and female neurons, *F*_CRF_(1, 24) = 38.82, *P* < 0.0001. **G** Representative family of 1 s hyperpolarizing and depolarizing current injections used characterize passive membrane properties and spike rate in aCSF (gray) and after 50 nM CRF (blue). **H** Example steady-state current–voltage dependence plot. Input resistance was determined by linear fit and slope at 0pA and deviation from fit indicates rectification. **I** CRF reduced membrane input resistance in male and female neurons, *F*_CRF_(1, 24) = 5.985, *P* = 0.022; this effect appeared most robustly in males. **J** CRF increased rectification of membrane potential in males and females, *F*_CRF_(1, 24) = 35.28, *P* < 0.0001. **K** CRF did not alter firing rates in response to 1 s depolarizing current injections in either males or females. Bar graphs indicate mean with individual replicates, line graphs mean (±SEM). *P < 0.05, **P < 0.01, ***P < 0.001, ****P < 0.0001 (Sidak’s tests).

Active and passive intrinsic properties (Supplementary Table 1) were computed from insular pyramidal neurons before and after the application of CRF (50 nM). Each parameter was analyzed for sex differences, CRF effects and sex by CRF interactions. With regard to sex differences in intrinsic properties, male and female intrinsic properties under aCSF recording conditions were comparable; no main effects of sex were present for any parameter. CRF altered many intrinsic properties including depolarization of resting membrane potential (Fig. 1A), reduction in action potential (AP) amplitude and rise rate (Fig. 1B), and corresponding increase in AP half-width (Fig. 1C). Many insular cortex pyramidal neurons have a bursting phenotype [29] and CRF appeared to reduce after depolarization (ADP) amplitude (Fig. 1D) and increased the current required to elicit a burst (burst ratio, Fig. 1E). CRF reduced firing rate (Table 1) consistent with CRF effects in the hippocampus [35]. A few sex-specific effects of CRF emerged in the passive properties. In males, CRF reduced input resistance (Fig. 1F) and in females, CRF reduced the membrane time constant. Interestingly, CRF increased the rectification ratio (Fig. 1G) to a similar extent in males and females. These results are not likely the result of diffusion or cell dialysis effects during recordings because under identical circumstances and recording times, these parameters are stable [29]. To establish whether the effects of CRF on insular cortex pyramidal neurons were mediated by action at the CRF_1_ receptor, which is the primary CRF receptor in insula [16, 17], we replicated the intrinsic characterization (Supplementary Fig. 1). For each parameter that was significantly changed by CRF in the first experiment, the CRF_1_ antagonist appeared to prevent those changes suggesting that the CRF_1_ receptor is the primary target of CRF in the insular cortex.

**Table 1:** Effect of 50 nM CRF on intrinsic properties of insular cortex pyramidal neurons.

Parameter	Sex	Means (SEM)		Results		Summary
		aCSF	CRF	Main effects	Interaction	
Membrane potential (mV)	M	−66.65 (1.527)	−59.64 (2.007)	CRF, *F*_1,24_ = 72.93, ***P*** **<** **0.0001**	*F*_1,24_ = 5.124, ***P*** **=** **0.033**	⇧
	F	−68.41 (1.189)	−64.33 (1.268)	Sex, *F*_1,24_ = 2.267, *P* = 0.145		⇧
Rheobase (pA)	M	1256.42 (105.92)	1226.42 (129.94)	CRF, *F*_1,24_ = 0.282, *P* = 0.600	*F*_1,24_ = 0.924, *P* = 0.346	=
	F	1515.00 (132.76)	1619.17 (157.49)	Sex, *F*_1,24_ = 3.573, *P* = 0.070		=
Spike amplitude (mV)	M	73.35 (3.852)	70.99 (4.039)	CRF, *F*_1,24_ = 4.933, ***P*** **=** **0.036**	*F*_1,24_ = 0.263, *P* = 0.612	⇩
	F	80.58 (3.764)	76.80 (4.136)	Sex, *F*_1,24_ = 1.430, *P* = 0.243		⇩
AP rise rate (mV/ms)	M	181.1 (17.55)	157.6 (14.548)	CRF, *F*_1,24_ = 18.93, ***P*** **=** **0.0002**	*F*_1,24_ = 0.535, *P* = 0 .472	⇩
	F	229.2 (25.536)	196.2 (26.136)	Sex, *F*_1,24_ = 2.253, *P* = 0.146		⇩
AP half-width (ms)	M	1.248 (0.075)	1.357 (0.064)	CRF, *F*_1,24_ = 16.69, ***P*** **=** **0.0004**	*F*_1,24_ = 0.134, *P* = 0.717	⇩
	F	1.125 (0.074)	1.256 (0.098)	Sex, *F*_1,24_ = 1.121, *P* = 0.300		⇩
After depolarization (mV)	M	10.36 (1.365)	8.593 (1.312)	CRF, *F*_1,24_ = 26.83, ***P*** **<** **0.0001**	*F*_1,24_ = 2.142, *P* = 0.156	⇩
	F	12.98 (0.805)	9.808 (0.688)	Sex, *F*_1,24_ = 1.554, *P* = 0.225		⇩
Burst ratio	M	0.2608 (0.052)	0.3889 (0.055)	CRF, *F*_1,21_ = 38.82, ***P*** **<** **0.0001**	*F*_1,21_ = 3.769, *P* = 0.066	⇧
	F	0.2153 (0.032)	0.2825 (0.024)	Sex, *F*_1,21_ = 1.600, *P* = 0.220		⇧
Input resistance (MΩ)	M	130.9 (15.318)	120.9 (14.663)	CRF, *F*_1,24_ = 5.985, ***P*** **=** **0.0221**	*F*_1,24_ = 2.497, *P* = 0.127	⇩
	F	104.9 (13.460)	102.8 (14.249)	Sex, *F*_1,24_ = 1.156, *P* = 0.293		=
Time constant (ms)	M	21.71 (0.946)	20.07 (0.946)	CRF, *F*_1,24_ = 12.37, ***P*** **=** **0.0018**	*F*_1,24_ = 0.173, *P* = 0.681	=
	F	24.25 (0.799)	22.17 (0.726)	Sex, *F*_1,24_ = 4.235, *P* = 0.051		⇩
Rectification ratio (mV)	M	0.8343 (0.023)	0.9014 (0.023)	CRF, *F*_1,24_ = 35.28, ***P*** **<** **0.0001**	*F*_1,24_ = 0.611, *P* = 0.442	⇧
	F	0.8158 (0.032)	0.9033 (0.025)	Sex, *F*_1,24_ = 0.058, *P* = 0.810		⇧
Sag ratio	M	1.359 (0.175)	1.384 (0.181)	CRF, *F*_1,24_ = 1.339, *P* = 0.259	*F*_1,24_ = 2.072, *P* = 0.163	=
	F	1.555 (0.244)	1.331 (0.231)	Sex, *F*_1,24_ = 0.065, *P* = 0.801		=
sAHP (mV)	M	−655.21 (85.958)	−765.43 (84.764)	CRF, *F*_1,24_ = 3.080, *P* = 0.092	*F*_1,24_ = 0.517, *P* = 0.479	=
	F	−659.41 (49.605)	−705.58 (44.492)	Sex, *F*_1,24_ = 0.092, *P* = 0.479		=
Firing rate (spikes/s @250 pA)	M	57.214 (17.354)	51.143 (19.000)	^a^CRF, *F*_1,24_ = 9.611, ***P*** **=** **0.005**	^a^*F*_1,24_ = 0.440, *P* = 0.503	⇩
	F	52.092 (16.433)	38.050 (14.649)	^a^Sex, *F*_1,24_ = 0.010, *P* = 0.921		=

Data were analyzed with a two-way analysis of variance. Summary indicates the effect of CRF and sex on parameter measured. Arrows indicate significant differences between aCSF and CRF conditions (*P*s < 0.05, Sidak-corrected *t*-test).

^a^The distribution of firing rates were negatively skewed so the data were converted with a log transform prior to analysis with ANOVA.

### CRF augments excitatory neurotransmission in the insula

**Fig. 2:**
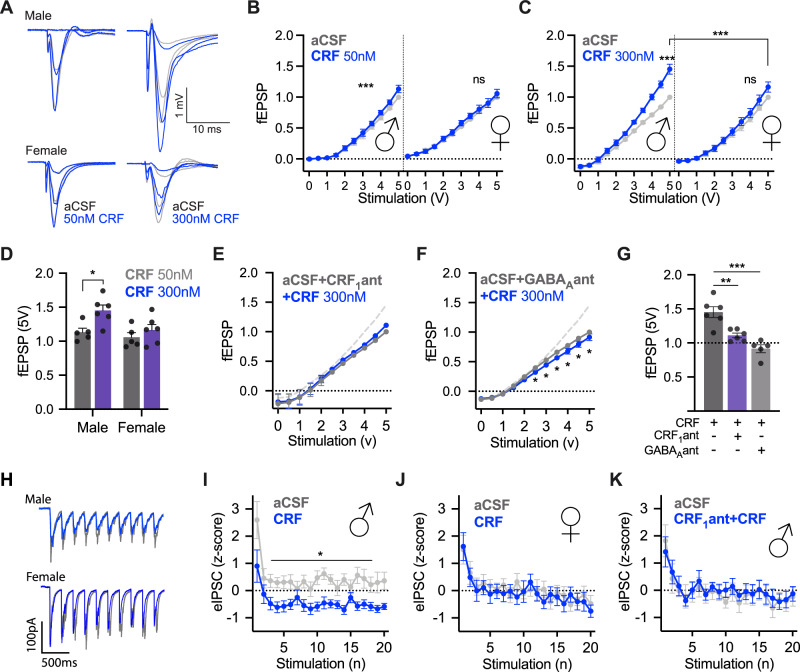
CRF has dose- and sex-dependent synaptic effects in insular cortex slices. **A** Representative traces of male (above) and female (below) field excitatory postsynaptic potentials (fEPSP) at 1, 3 and 5 V under aCSF (gray) and after 50 nM (left, blue) or 300 nM CRF (right, blue) conditions. For analysis, traces were normalized to the peak amplitude of the fEPSP evoked at 5 V in aCSF. **B** Bath application of 50 nM CRF significantly increased fEPSPs in male insular cortex slices in biphasic 0–5 V I/O curves *F*_Voltage × CRF_ (20, 80) = 5.791, *P* < 0.0001 with post hoc tests showing CRF being significantly increased over baseline at 3 V (*P* = 0.0425), 3.5 V (*P* < 0.0001), 4 V (*P* = 0.0001), 4.5 V (*P* = 0.0091) and 5 V (*P* = 0.0009). However, there was no significant effect of CRF on female slices *F*_Voltage × CRF_ (20, 80) = 0.5351, *P* < 0.5667. A three-way ANOVA revealed significant interactions between voltage and CRF and sex: *F*_Voltage × CRF_(10, 80) = 3.654, *P* = 0.0005, *F*_Voltage × Sex_(10, 80) = 2.910, *P* = 0.0037 as well as a main effect of sex, *F*_Sex_(1, 8) = 10.53, *P* = 0.0118. **C** Bath application of 300 nM CRF led to a sex difference in fEPSPs such that males showed increased synaptic efficacy but not females resulting in a significant three-way interaction, *F*_Voltage × Sex × CRF_ (10, 100) = 5.306, *P* < 0.0001. Males showed significant increases in fEPSP under CRF conditions via Tukey’s multiple comparison tests at 2.5 V (*P* = 0.0370), 3 V (*P* = 0.0212), 3.5 V (*P* = 0.0124), 4 V (*P* = 0.0063), 4.5 V (0.0040) and 5 V (*P* = 0.0058). **D** Comparing 5 V responses (normalized to female aCSF 5 V) under 50 nM versus 300 nM CRF by sex revealed main effects of sex, *F*_Sex_(1, 18) = 5.737, *P* = 0.0277, and CRF, *F*_CRF_(1, 18) = 7.855, *P* = 0.0118. Sidak’s post hoc tests showed that there was a significant dose effect in males *t*(18) = 2.981, *P* = 0.0471, but not in females *t*(18) = 0.9826, *P* = 0.9165. **E** CRF_1_ antagonist CP154526 (10 μm) coapplied with 300 nM CRF prevented CRF from increasing fEPSPs in slices from male rats. The dashed gray line depicts the effect of 300 nM CRF alone for comparison. While there was a significant interaction *F*_Voltage × CRF_(20, 89) = 3.276, *P* < 0.0001. There was no main effect of CRF, *F*_CRF_(2, 10) = 0.0362, *P* = 0.9646. No post hoc comparisons were significant across treatments at different voltages. **F** Coapplication of the GABA_A_ antagonist SR95531 prevented the enhancing effect of 300 nM CRF and led to a significant decrease in fEPSPs *F*_Voltage × CRF_(10, 40) = 3.464, *P* = 0.0024 in slices from male rats. Significant Tukey’s post hoc comparisons were found at 2.5 V (*P* = 0.0422), 3 V (*P* = 0.0144), 3.5 V (*P* = 0.0038), 4 V (*P* = 0.0026), 4.5 V (*P* = 0.0014), and 5 V (*P* = 0.0116). **G** 5 V fEPSPs after CRF, CRF + CP154526 and CRF + SR95531 (300 nM) were normalized to the relative 5 V fEPSP in aCSF in (**D**) to summarize the effect of CRF_1_ and GABA_A_ receptor antagonist on fEPSP. Both CRF_1_ antagonist and GABA_A_ antagonist prevented the increase in fEPSP caused by CRF, *F*(2, 14) = 19.42, *P* < 0.0001. Tukey’s post hoc tests show a significant difference between CP154526 + CRF and 300 nM CRF (*P* = 0.0031) and SR95531 + CRF and 300 nM CRF (*P* < 0.0001). **H** Voltage-clamp recordings of evoked inhibitory postsynaptic currents (eIPSC) from deep layer insular cortex pyramidal neurons under baseline (gray-aCSF with glutamatergic synaptic antagonists) and after 50 nM CRF (blue) in slices from male or female rats. Twenty eIPSCs were evoked by extracellular bipolar electrodes at 5 Hz (the first 10 are shown). For analysis, eIPSC amplitudes were normalized using z-scores computed from the mean and standard deviation of the aCSF recordings (panels **I**, **J**). Basal eIPSC amplitudes did not differ between male and female recordings. **I** CRF significantly reduced the amplitude of eIPSCs in males, *F*_CRF_ (1, 8) = 7.006, *P* = 0.0294. **J** CRF did not alter eIPSC amplitudes in females *F*_CRF_(1, 8) = 0.0531, *P* = 0.8235. **K** Pretreatment of the slice with CRF_1_ antagonist eliminated the effect of CRF on eIPSCs in slices from male rats, *F*_CRF_(1, 7) = 0.0547, *P* = 0.8218.

The effects of CRF on intrinsic properties suggest a mix of augmentation (depolarization) and dampening (reduced AP parameters, reduced bursting) modulation. To better understand how CRF might alter insular cortex information throughput, we investigated CRF effects on synaptic transmission (Fig. 2A). fEPSP input/output curves were generated in aCSF and then again in either 50 or 300 nM CRF from stimulation within the insular cortex. In male, but not female, slices, CRF caused a dose-dependent leftward shift indicating augmented synaptic efficacy at both 50 nM (Fig. 2B) and 300 nM (Fig. 2C, D). We then tested whether CRF augmented insular synaptic excitability in males is CRF_1_ dependent by coapplying 300 nM CRF and 10 μM CP154526 during I/O curves. The CRF_1_ antagonist blocked the effects of CRF on fEPSP (Fig. 2E). In summary, CRF application increased excitatory synaptic efficacy in the insula of male rats via action at the CRF_1_ receptor.

### CRF reduces evoked presynaptic GABA release

An enhancement of fEPSP transmission by CRF could result from direct augmentation of glutamatergic transmission or from inhibitory modulation of GABAergic transmission. Because CRF depolarized principle glutamatergic neurons we first tested whether CRF application would augment glutamatergic transmission by recording spontaneous EPSCs and AMPA and NMDA mediated currents before and after CRF (50 nM). Surprisingly, CRF had no effect on either measure or the AMPA/NMDA ratio (Supplementary Fig. 2). Therefore, we hypothesized that the enhancement of fEPSP observed after CRF was dependent upon GABA_A_. We tested this by pretreating male insular slices with GABA_A_ antagonist SR95531 (2 μM) and then applied CRF (300 nM) and repeated fEPSP input/output characterization. In the presence of the GABA_A_ antagonist, CRF had no apparent effect (Fig. 2F). The same pattern was observed when using picrotoxin, a naturally occurring GABA_A_ antagonist (data not shown). In a direct comparison, we found both CRF_1_ antagonist and the GABA_A_ antagonist blocked CRF based increases in insular excitatory synaptic transmission (Fig. 2G).

Because it is possible that CRF acts directly on GABAergic neurons in addition to its effects on principle neurons, we next used whole-cell, voltage-clamp recordings to investigate the effect of CRF on eIPSCs (Fig. 2H). Here, CRF caused a marked reduction in eIPSC amplitude in males (Fig. 2I) but the effect was not present in females (Fig. 2J). Furthermore, pretreatment with the CRF_1_ antagonist eliminated the inhibitory effect of CRF on eIPSCs in males (Fig. 2K). In a separate experiment, we examined spontaneous IPSCs and found reduced IPSC frequency, a trend consistent with CRF negatively modulating GABAergic tone (Supplementary Fig. 2).

### Insular CRF increases social exploration of conspecifics in males but not females

**Fig. 3:**
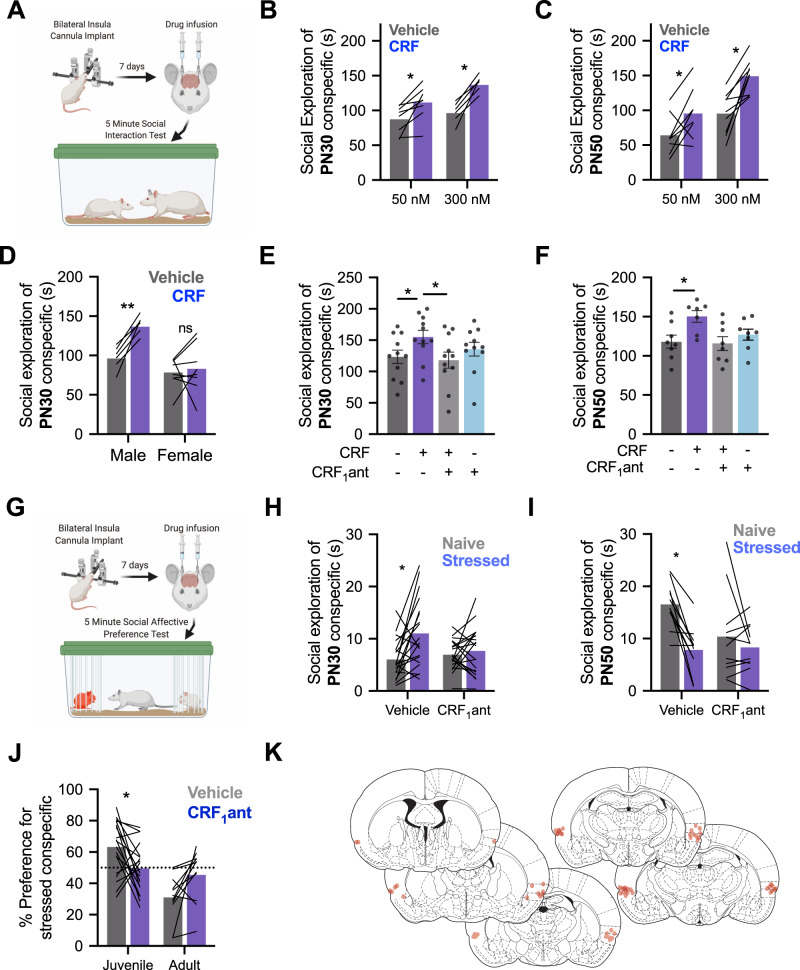
CRF augments social behavior and is necessary for social affective behavior. **A** A diagram laying out the experimental procedure for social exploration tests. Cannula was placed in the insular cortex. On the day of testing, rats were given 1 h to acclimate to the testing cage. CRF or saline vehicle infusions were made 40 min prior to social interaction with a juvenile (P30) or adult (P50) conspecific for 5 min. **B** In male rats, CRF increased social exploration of juvenile conspecifics, *F*_CRF_(1, 13) = 48.5, *P* < 0.0001. Sidak’s post hoc tests revealed significantly increased social exploration at both 50 nM (*P* = 0.0044) and 300 nM (*P* < 0.0001). **C** In male rats, CRF also increased social exploration of P50 conspecifics, *F*_CRF_(1, 15) = 24.99, *P* = 0.0002. Sidak’s post hoc tests showed that social exploration was increased at both 50 nM (*P* = 0.0450) and 300 nM (*P* = 0.0007) doses. **D** In female rats, 300 nM CRF did not alter social interaction with juvenile conspecifics. Female data were compared males at 300 nM (data replotted from panel **B** to facilitate comparison) revealing a sex-specific effect of CRF *F*_Sex × CRF_(1, 13) = 6.517, *P* = 0.0241 such that males showed increased social exploration following CRF treatment (*P* = 0.0033) but females did not (*P* = 0.8615). **E** Social exploration by males (*n* = 11) of juvenile (PN30) conspecifics was altered by CRF and the CRF_1_ antagonist CP154526. A two-way repeated-measures ANOVA revealed a significant interaction *F*_CRF × CRF1antagonist_(1, 10) = 12.82, *P* = 0.005. 300 nM CRF increased social exploration that was significantly greater than both the vehicle condition (Tukey’s post hoc test, *P* = 0.0359) and the combined CRF and CRF_1_ antagonist condition (*P* = 0.0152). Independently, the CRF_1_ antagonist had no effect on social exploration (*P* = 0.6093). **F** In tests of male rats (*n* = 8) with adult conspecifics, CRF_1_ antagonist blocked the increase in social interaction caused by CRF, *F*_CRF × CRF1antagonist_(1, 6) = 12.67, *P* = 0.0119. Mean social interaction time was greatest in the group that received CRF alone that differed from the vehicle (*P* = 0.0142) and combined CRF and CRF_1_ antagonist conditions (*P* = 0.0457). **G** Diagram of the social affective behavior test (SAP) paradigm. Rats received insular cannula implants. On the test day, drug infusions were made 40 min before SAP tests consisting of a 5-min interaction with a naive and stressed same-sex conspecific. The amount of time spent socially investigating each conspecific is recorded. **H** When tested under vehicle conditions with PN30 conspecifics, male rats (*n* = 19) exhibit greater exploration of the stressed rat (*P* = 0.0027); this pattern was blocked by the CRF_1_ antagonist (*P* = 0.8293) supported by a significant interaction, *F*_CRF1antagonist × Stress_(1, 18) = 5.225, *P* = 0.0346. **I** Experimental male rats (*n* = 11) spent less time interacting with stressed PN50 adult conspecifics in the vehicle condition but this pattern was blocked by the CRF_1_ antagonist, *F*_CRF1antagonist × Stress_ (1,10) = 6.133, *P* = 0.0327. Post hoc comparisons revealed a preference for more interaction with naive adults in vehicles (*P* = 0.0020) but no difference with the CRF_1_ antagonist (*P* = 0.5_1_50). **J** For comparison, time spent interacting with naive and stressed conspecifics from panels **H** and **I** was converted to a preference score (% preference = time investigating stressed conspecific/total investigation time × 100). In vehicle conditions, experimental rats preferred interaction with stressed juveniles, but avoided stressed adults and CRF_1_ antagonist treatment appeared to reduce these preferences, *F*_Age × Drug_ (1, 28) = 11.30, *P* = 0.0023. When comparing juveniles, the percent preference for the stressed conspecific was significantly reduced by CRF_1_ antagonist (*P* = 0.0227). When comparing adults, although the CRF_1_ antagonist appeared to eliminate the preference for naive conspecifics, the Sidak-corrected post hoc test did not reach significance (*P* = 0.0768). **K** Cannula maps showing the placement of in-dwelling cannula across all experiments related to Fig. 3. Diagrams in panels **A** and **G** were created with BioRender.com.

The foregoing physiological findings suggest that CRF augments synaptic transmission in IC via downregulation of local GABAergic interneurons. Manipulations that alter insular excitability are associated with changes in social affective behaviors [29, 32, 36, 37] and the CRF system is implicated in many aspects of social behavior [15]. To begin to assess whether the effect of CRF in the slices was behaviorally relevant, we utilized social interaction behavior tests. We first investigated whether CRF influenced social investigation toward juvenile (P28) or adult (P50) conspecifics in male rats in a 5-min social investigation test (Fig. 3A). Bilateral infusion of either 50 nM (238 pg/500nL) or 300 nM (1.4 ng/500 nL) doses of CRF to the insula of males led to an increase in the social investigation of both juvenile (Fig. 3B) and adult (Fig. 3C) conspecifics compared to social investigations following saline injections. To establish whether the sex difference observed in physiology was present in social behavior, a separate cohort of female rats received social interaction tests with juveniles following a 300 nM injection of CRF. This dose was chosen because it showed a robust effect in male slices while female slices showed no response to either 50 or 300 nM doses. Consistent with physiology, CRF did not alter female social interaction (Fig. 3D).

Next, we tested whether augmentation of male social interaction by CRF was CRF_1_ receptor dependent. As above, CRF increased social interaction compared to saline or CP154526, which was without effect (Fig. 3E, F). Importantly, social interaction levels did not differ from saline control levels when CRF was coadministered with CP154526 showing that CRFs augmentation of social behavior relies on CRF_1_ receptor activation in the insula.

### Social investigation of stressed conspecifics requires insular CRF_1_

The previous gain-of-function results provide evidence that insular CRF and CRF_1_ may contribute to social behavior. In a seminal study, Sterley et al. [12] demonstrated in mice that the CRF system is engaged during social encounters with stressed conspecifics. Similarly, we demonstrated that insula activity determines the nature of social interaction with stressed conspecifics, either approach or avoidance, in a SAP test [29, 32]. We predicted that, in male rats, exposure to a stressed conspecific would evoke insular cortex CRF release that, via CRF_1_ may contribute to social affective behavior (Fig. 3G). CRF_1_ blockade prevented the formation of a preference for stressed juveniles (Fig. 3H) such that approach behavior did not differ toward stressed or unstressed conspecifics. After vehicle injections, rats displayed a preference for unstressed adult conspecifics but this preference was eliminated via injection of CRF_1_ antagonist (Fig. 3I). Percent preference for stressed individuals was significantly altered by CRF_1_ antagonist injections such that test rats lost preference for both stressed juveniles and non-stressed adults (Fig. 3J). To test whether CRF contributes to non-social emotional states associated with threat and are mediated by posterior insula [9] we injected CRF to the insula in rats during threat learning or threat recall in a Pavlovian conditioning procedure that results in an insula-dependent conditioned freezing response [38], but CRF had no effect (Supplementary Results and Supplementary Fig. 3).

### CB_1_ is necessary for the augmentation of excitatory neurotransmission and social behavior increases caused by CRF

**Fig. 4:**
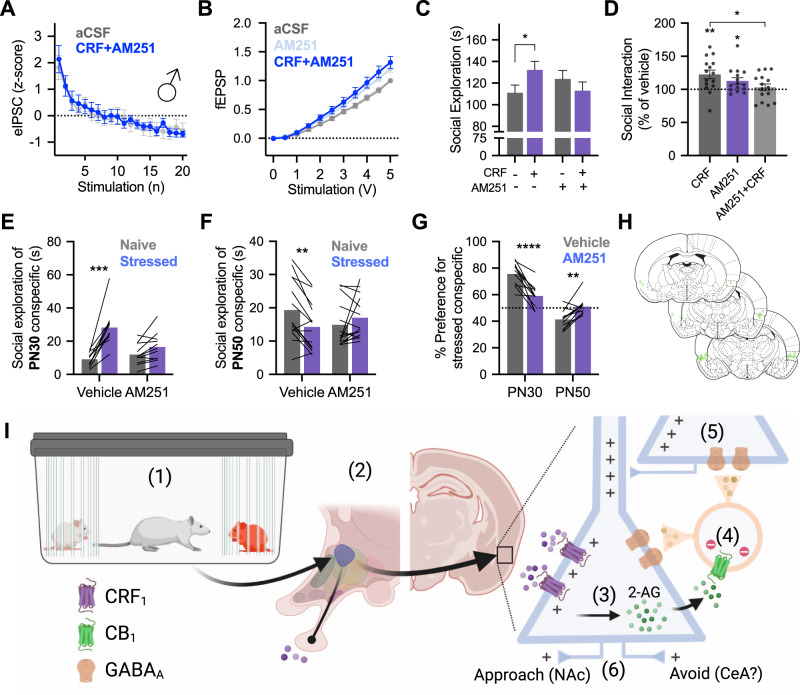
Cannabinoid 1 receptor is necessary for the behavioral and synaptic effects of insular CRF. **A** fEPSPs recorded from insular cortex slices (*n* = 8) were insensitive to 300 nM CRF when applied with CB_1_ receptor antagonist AM251 (2 μM), *F*_Voltage × Drug_ (1,11) = 2.992, *P* = 0.0959. **B** In voltage-clamp recordings of insular cortex pyramidal neurons (*n* = 9), AM251 prevented the inhibition of eIPSCs previously caused by CRF (50 nM) application, *F*_Drug_(1, 8) = 0.0157, *P* = 0.9032. **C** In a 5-min social interaction test with male rats (*n* = 15) CRF injected into the insular cortex increased exploration of juvenile conspecifics (*P* = 0.0270). CRF given in combination with AM251 did not increase social exploration, *F*_CRF × AM251_ (1, 14) = 9.102, *P* = 0.0092. **D** For comparison, raw social interaction times from panel **C** are shown as percent of time relative to the no drug condition. CRF increased interaction (one-sample *t*-test compared to 100%, *t*(14) = 3.422, *P* = 0.0041, AM251 alone increased interaction, *t*(14) = 2.620, *P* = 0.0202, but CRF given with AM251 did not differ from vehicle levels, *t*(14) = 0.5935, *P* = 0.5623. Importantly, social interaction was greater with CRF alone than in combination with AM521, (*P* = 0.0110, Sidak test after significant one-way ANOVA, *F*(2, 28) = 3.705, *P* = 0.0374). **E** In SAP tests with juveniles (*n* = 11), AM251 prevented the formation of a preference for stressed juvenile conspecifics, *F*_Drug × Stress_(1, 9) = 22.53, *P* = 0.0010, such that the preference for stressed juveniles present during vehicle testing (*P* < 0.0001) was eliminated during AM251 testing (*P* = 0.0940). **F** In SAP tests with adult conspecifics (*n* = 16), AM251 eliminated the preference of test rats for naive adult *F*_Drug × Stress_(1, 13) = 19.93, *P* = 0.0006. A significant preference for naive adults was present in vehicle test rats (*P* = 0.0013) that was not present in AM251 rats (*P* = 0.1691). **G** Percent preference for stressed conspecifics was significantly altered by a combination of AM251 treatment and age of conspecific *F*_Age × Drug_(1, 20) = 43.12, *P* < 0.0001. Specifically, the percent preference for stressed juveniles was significantly reduced (*P* < 0.0001) while the percent preference for stressed adults was significantly increased (*P* = 0.0054). **H** Cannula placements of all animals in the experiments contained in Fig. 4. **I** Summary of behavioral pharmacology. (1) Exposure to the stressed conspecific causes emotion transfer and release of CRF (purple) from the paraventricular nucleus of the hypothalamus. (2) CRF binds to principal neurons in the insular cortex leading to depolarization and (3) synthesis of endocannabinoids, such as 2-AG. (4) Endocannabinoids (green) bind to CB_1_ receptors on presynaptic GABAergic interneurons (orange) leading to hyperpolarization and suppression of local inhibition. (5) The loss of GABAergic inhibition permits greater excitatory synaptic transmission among principal neurons (6) whose output shapes social approach or avoidance by projections to nodes in the social decision-making network. Diagram created in BioRender.com. Bar graphs indicate mean with individual replicates, line graphs mean (±SEM). *P < 0.05, **P < 0.01, ***P < 0.001, ****P < 0.0001 (Sidak’s tests).

To better resolve the mechanism by which CRF alters insular excitatory/inhibitory tone we considered two possible mechanisms. First, CRF might directly alter GABAergic interneurons, GABA release, or GABA_A_ kinetics. Second, CRF might indirectly modify GABA function via actions that begin with the CRF_1_ receptor on pyramidal neurons. While the former remains interesting, there are technical challenges with direct assessment of interneuron function in rats. Regarding the latter, depolarization and accumulation of intracellular calcium leads to the synthesis and retrograde release of endocannabinoids. Via action at the G_i_-protein coupled presynaptic cannabinoid receptor 1 (CB_1_), depolarization leads to the reduction in GABA release and the phenomenon of DSI [24]. Because CRF depolarized pyramidal neurons, we hypothesized that CRF might indirectly affect presynaptic GABA tone via endocannabinoids which led us to consider the role of CB_1_ in mediating effects of CRF. We utilized AM251 (2 μM), a CB_1_ inverse agonist [39], in fEPSPs, eIPSCs and social behavior in male rats. Coapplication of AM251 prevented CRF effects on synaptic transmission (Fig. 4A). In whole-cell recordings of eIPSCs, CRF had no effect when administered with AM251 (Fig. 4B). As before, insular CRF injections increased social interaction with juvenile conspecifics. Interestingly, AM251 itself increased both fEPSP amplitude and social exploration which might be due to the blockade of CB_1_ receptors on both principle neurons and inhibitory interneurons resulting in a net excitatory modulation. AM251 (Fig. 4C) prevented insular CRF from increasing social investigation of a juvenile conspecific and brought the total amount of social interaction to vehicle levels (Fig. 4D). In SAP tests, AM251 prevented the formation of a preference for stressed juveniles (Fig. 4E) or unstressed adult conspecifics (Fig. 4F) indicating that, in addition to CRF_1_, CB_1_ is necessary for the evaluation of these socioemotional cues (Fig. 4G).

### Sex differences exist in CRF_1_ and CB_1_ mRNA expression and cellular distribution in the insular cortex

**Fig. 5:**
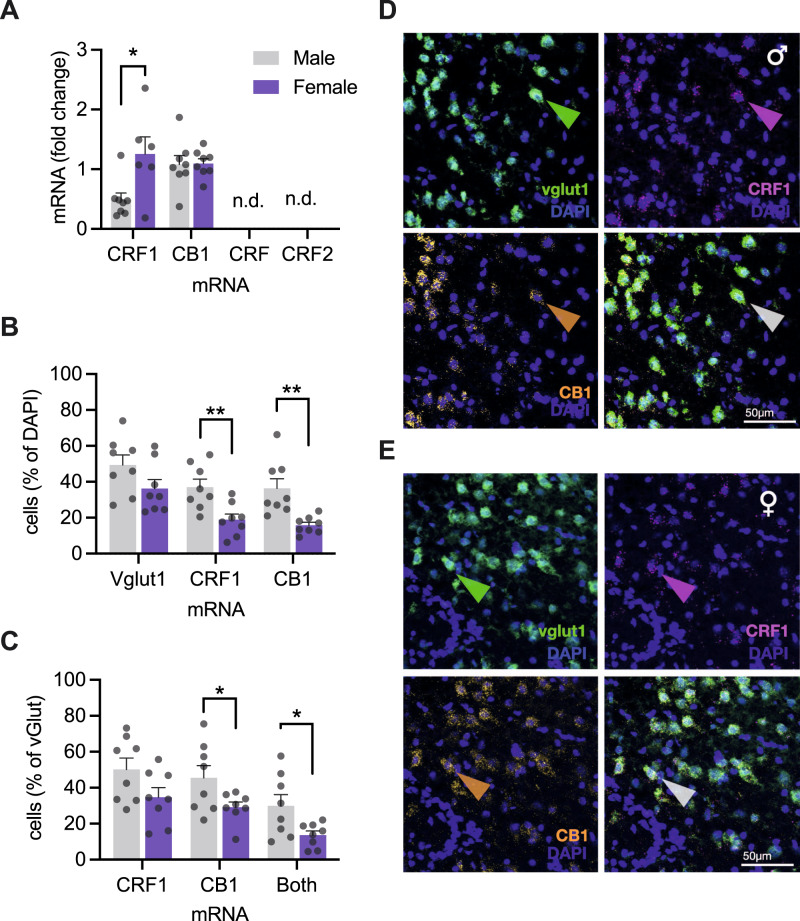
Cellular distribution of CRF_1_ and CB_1_ receptor mRNA in the insular cortex. **A** qPCR analysis of relative mRNA expression revealed greater CRF_1_ mRNA in females compared to males *t*(12) = 2.728, *P* = 0.183, and equal CB_1_ mRNA across sexes *t*(14) = 0.090, *P* = 0.929. **B** RNAScope was performed for CRF_1_, CB_1_, and vglut1 mRNAs. Fluorescent grains were counted in the left and right hemispheres of the posterior insular cortex. The total number of cells was determined by counting DAPI nuclei in each hemisphere. Nuclei containing 3 or more fluorescent grains were considered mRNA expressing cells and shown as the % of the total cells. The number of vglut1 cells was equal between sexes, but the portion of cells expressing CRF_1_ *F*_Sex_(1, 14) = 11.19, *P* = 0.005, or CB_1_, F_Sex_(1, 14) = 13.07, *P* = 0.003, mRNA was approximately double in males compared to females. **C** Looking at expression of CRF_1_ and CB_1_ mRNA in vglut1 cells, shown as a percent of the total vglut1 cells per hemisphere, male rat sections contained more CB_1_ mRNA expressing vglut cells, and more vglut cells expressing both CB_1_ and CRF_1_ mRNA, *F*_Sex_(1, 14) = 4.489, *P* = 0.044). The number of vglut cells expressing CRF1 mRNA was greater on average in males than females, but did not reach significance *F*_Sex_(1, 14) = 3.944, *P* = 0.067. In males, there were more vglut cells colocalized with both CRF_1_ and CB_1_, *F*_Sex_(1, 14) = 6.576, *P* = 0.023. **D**, **E** Representative digital photomicrographs of RNAScope in situ hybridization and fluorescent visualization of DAPI and vesicular glutamate transporter 1 (*vglut1)*, CRF_1_ (crhr1), and CB_1_ (cn1r) mRNA from male (**A**) and female (rats) insular cortex coronal sections (20 um, *n* = 8/sex). Sections were selected from subjects nearest to the mean values for CRF_1_ + DAPI colabeling. Colored arrowheads indicate cells with coexpression of all three mRNAs with DAPI. Bar graphs indicate mean (±SEM) with individual replicates. *P < 0.05, **P < 0.01, ***P < 0.001, ****P < 0.0001 (Sidak’s tests).

The pattern of sex-specific effects of CRF on physiology and social behavior led us to hypothesize that sex differences exist in the amount or distribution of insular CRF_1_ and CB_1_. Using qPCR on insular cortex micro punches we found greater CRF_1_ mRNA in female rats compared to males (Fig. 5A). CRF (*crh*) and CRF type 2 receptor (*crhr2*) mRNAs were not detectable, which is consistent with prior reports [40, 41]. To understand the functional sex differences we turned to RNAScope fluorescent in situ hybridization, to visualize and colocalize mRNAs of glutamatergic neurons (*vGlut1*), CRF_1_ (*crhr1*) and CB_1_ (*cnr1*) in insula sections from male and female adult rats (Fig. 5D, E). Males had more DAPI nuclei labeled with CRF_1_ and CB_1_ mRNA (Fig. 5B). Looking at putative glutamate neurons (vGlut1 positive cells, Fig. 5C), there was a trend for greater CRF_1_ in males, but this did not reach significance (*P* = 0.067). However, in males we found more glutamate neurons colocalized with CB_1_ and more glutamate neurons colocalized with both CRF_1_ and CB_1_ mRNAs than females.

## Discussion

We investigated neural mechanisms by which social stress signals influence social behavior. We focused on the neuropeptide CRF, which is released during social interactions with stressed conspecifics [12], and the posterior insular cortex (IC), a structure needed for social affective behavior [29]. Electrophysiology, pharmacology, behavior and molecular experiments revealed a sex-specific role for CRF as a modulator of insular synaptic physiology and social behavior. In males only, CRF caused a reduction in presynaptic inhibitory tone, likely via release of retrograde endocannabinoids acting at presynaptic CB_1_ receptors. Behaviorally, insular CRF injections increased social interaction, and CRF_1_ and CB_1_ receptor antagonists both interfered with behavior in a SAP test. To understand the basis for the sex difference we used qPCR and in situ hybridization to describe the relative expression and cellular distribution of CRF_1_ and CB_1_ mRNAs. Although we found more overall CRF_1_ mRNA in females, we found that males had more CRF_1_ positive cells and increased CRF_1_ and CB_1_ colocalization on glutamate neurons. In sum, these results add to our understanding of CRF as a neuromodulator, integrate CRF into a social affective behavior process, and have important implications for understanding the neurobiology of social cognition.

The depolarizing effect of CRF on membrane potential is consistent with the G_S_-protein signaling and cAMP modulation of cation channels [42]. However, the dampening effects of CRF on the AP, reduction of ADP and the increase in rectification ratio suggest additional effects of CRF via modulation of ion channels such as T-type voltage-gated calcium channels [43] or voltage-gated potassium channels [35]. The most robust effect of CRF was depolarization of the membrane potential which we predicted would lead to augmentation of excitatory synaptic transmission. When looking at synaptic measures, however, sex-specific effects of CRF become clear. In males, CRF augmented fEPSPs in a dose- and CRF_1_-dependent fashion. The synaptic effect of CRF also depended upon GABA_A_ receptors, suggesting a more complex mechanism of action for CRF than direct modulation of principal neurons via CRF_1_. We further investigated this in eIPSCs as a measure of GABAergic tone and found a CRF_1_-dependent reduction in eIPSC amplitude in males, but not females. Because depolarization alone is sufficient to inhibit presynaptic GABAergic transmission via retrograde endocannabinoids and the CB_1_ receptor [24] we tested the dependence of CRF effects on CB_1_ and found that both the augmentation of fEPSPs and suppression of eIPSCs were prevented by pretreatment with a CB_1_ receptor inverse agonist. These results suggest that CRF action within the insula may begin with CRF_1_-mediated depolarization of pyramidal neurons that then release endocannabinoids which, in turn, cause suppression of presynaptic GABAergic neurons accounting for the increase in excitatory transmission in male insula (Fig. 4I). This mechanism of CRF action is present in the lateral habenula [44]. Similarly, oxytocin can also trigger endocannabinoid release increasing postsynaptic excitability [45]. This may indicate that insular control of social behavior by different neuromodulators, oxytocin [29] and CRF is driven by a convergent mechanism whereby CB_1_-dependent inhibition of presynaptic GABA leads to postsynaptic excitability. One possibility for how this occurs is DSI and, interestingly, experimentally induced endocannabinoid-mediated DSI occurs after strong depolarization protocols [46], whereas here CRF led to depolarization of ~4–7 mV. It is also possible that eCBs synthesis and release were a result of G-protein mediated effects including the release of intracellular calcium or protein kinase-dependent actions that may occur downstream of insular CRF_1_ receptors [44]. Thus, important goals of future work include determining if small, sustained depolarizations are sufficient to evoke CB_1_-dependent presynaptic inhibition, identify the molecular cascades linking CRF and endocannabinoids (such as modulation of endocannabinoid metabolism as in [47, 48]), the type of presynaptic cells effected by endocannabinoids, the source of insular CRF, and the sex differences in these systems that result in male-specific effects.

Detecting socioemotional cues and using this information to inform whether to approach or avoid others is a vital aspect of social behavior. CRF receptors are distributed across many structures critical to social behavior [15] and the observer stress response, a form of emotion contagion, may be elementary to more complex social cognition that involves a distributed social affective behavior network [27]. Sterley et al. [9] observed that exposure to stressed conspecifics potentiated PVN CRF neurons. Here, insular injections of CRF increased social investigation of both juvenile and adult conspecifics, but only in male rats. In the SAP test, experimental rats typically prefer to interact with stressed juvenile conspecifics but avoid stressed adults [29, 33]. The CRF_1_ antagonist prevented this pattern suggesting that activation of the CRF acute stress response, as reported by Sterely et al., likely occurs in the experimental rat during the SAP test. The framework is that the decision to approach or avoid another involves assessment of the subject’s core affect (e.g., “Am I in danger?”) and social affective information about the social target (e.g., “Is the conspecific in danger?”). Avoidance occurs when the targets are adults, possibly because the subject experiences greater internal distress/danger as the adult targets could be communicating imminent environmental threat or pose a threat (e.g., aggression) themselves. Alternatively, when the targets are juveniles, there is less (or none) social threat and approach occurs, possibly because the signals given off by stressed juveniles are social attractants [49] or there are innate prosocial drives [50]. As this test is relatively new we have only examined a few additional circumstances but together they fit in this framework. First, the approach to the stressed adult occurs when the subjects are familiar [33] likely reflective of less subject anxiety due to familiarity. Second, another lab found that prior stress of the test subject led to social avoidance—interestingly also sensitive to manipulations of the peripheral stress system [51]. Third, adult rats avoid sick adults, a phenomenon that also requires insula [52]. Consistently, CRF mediates defensive responses to synthetic fox odor, a potent social stressor, by potentiation of prefrontal cortex excitatory synaptic transmission [26]. The current results suggest that CRF provides an organizing signal about the subject’s core affect.

The CRF system is complex, comprising of many sex, brain region and cell type-specific differences and functional contributions to behavior. As such, while the finding that CRF in the insula acts in a sex-specific manner to augment social behavior and drive social affective behavior in males is novel, the fact that a sex difference exists is not surprising. Here we began to understand the molecular basis for the sex difference by quantifying the relative expression and cellular distribution of CRF_1_ and CB_1_ mRNAs. Interestingly, qPCR revealed a greater relative amount of insular CRF_1_ mRNA in females compared to males and no difference in relative CB_1_ expression between males and females, indicating that the synaptic and behavioral sex differences found here are not likely due to less available CRF_1_. These results contrast some prior reports that find greater CRF_1_ mRNA in prefrontal cortex of males [53] but it is difficult to generalize across regions as CRF_1_ receptor binding varies in sex-specific ways with female adult rats tending to have more binding than males [54]. Regarding cellular distribution, we found more CRF_1_ and CB_1_ mRNA containing nuclei and glutamatergic neurons in males compared to females. While it is important to note that these measures of CRF_1_ and CB_1_ mRNAs are nuclear and may not reflect the actual expression of the receptors on synaptic terminals arriving from sources afferent to the insula, the distribution does illuminate a mechanism for sex-specific effects. First, the male insula contained more CRF_1_ expressing glutamatergic neurons than females, providing a larger population of neurons to be depolarized. Notably, both males and females exhibited CRF-induced changes in intrinsic neuronal physiology suggesting that female insula neurons express sufficient CRF_1_ for modulation of intrinsic excitability. Sex differences in physiology emerged at the synaptic level and the combination of mRNA distribution and pharmacology results suggest that this is due to much higher CB_1_ expression in males (Fig. 5 and Ref. [55]), which were required for the CRF-mediated effects on excitatory transmission, modulation of inhibitory presynaptic tone and social behavior. Thus, in males, CRF leads to a CRF_1_-dependent release of eCBs that modulate presynaptic inhibition and behavior via CB_1_ in males. Females may not have sufficient insular CB_1_ to complete this cascade of signals and so CRF does not alter synaptic efficacy or behavior. It remains possible that there are other sex differences including the distribution of these receptors within the cortical interneuron subtypes or the cellular signaling cascades involved, both of which are exciting and clinically relevant directions for future research.

Human neuroimaging studies implicate the insula in an impressive range of cognitive processes [2, 4, 5, 7, 8, 56], including emotion recognition [6], anticipatory fear and anxiety [46, 57, 58]. Acute stress increases insula activity and functional connectivity [59, 60] and trauma leads to lasting enhancement of processing within insula networks [11, 61]. In response to threat, CRF likely contributes to organizing insula and broader networks to determine what responses are situationally appropriate and the current results encourage further investigation of insular CRF in the stress-related psychopathologies.

## Supplementary information

Supplementary Methods and results

Supplementary Table 1
