# Supplementary material for: Insular cortex corticotropin-releasing factor integrates stress signaling with social affective behavior
Source: Neuropsychopharmacology. 2022 Feb 26;47(6):1156–68. doi: 10.1038/s41386-022-01292-7 (PMC9018766; doi:10.1038/s41386-022-01292-7)
Supplement: Supplementary file 1 — Supplementary Methods and results [file 41386_2022_1292_MOESM1_ESM.pdf]

Supplementary Methods

Animals

Male and Female Sprague-Dawley rats were obtained from Charles River Laboratories (Wilmington, MA) at either age PN45 (test rats and adult conspecifics) or PN21 (juvenile conspecifics). Testing occurred after a minimum of 7 days acclimation. Tissue for electrophysiology studies was obtained from adult rats from PN 52 to 70 days old. In behavioral studies, experimental rats were a minimum of 52 days old at the beginning of experiments, either behavioral tests or the implantation of cannula. Conspecific adult rats were used at 52-58 days old and conspecific juveniles were used at 28-31 days old. Rats were always group housed with 2-3 same-sex conspecifics and given access to food and water *ad libitum*. The vivarium was maintained on a 12 h light/dark cycle and all behavioral tests occurred within the first four hours of the light cycle. All procedures were conducted in accordance with the NIH *Guide for the care and use of laboratory animals* and approved by the Boston College Institutional Animal Care and Use Committee.

Electrophysiology

Solutions: Standard solutions were used for artificial cerebrospinal fluid (aCSF) and recording solutions were prepared as previously described (Sidiropoulou et al., 2009) and all reagents were purchased from ThermoFisher, Sigma or Tocris. aCSF recording composition was (in mM) NaCl 125, KCl 2.5, $NaHCO_3$ 25, NaH_2PO_4 1.25, $MgCl_2$ 1, $CaCl_2$ 2 and Glucose 10; pH = 7.40; 310 mOsm; aCSF cutting solution was: Sucrose 75, NaCl 87, KCl 2.5, $NaHCO_3$ 25, NaH_2PO_4 1.25, $MgCl_2$ 7, $CaCl_2$ 0.5, Glucose 25 and Kynureinic acid 1; pH=7.40, 312 mOsm. The internal recording solution consisted of (in mM) K^+-Gluconate: 115, KCl 20, HEPES 10, Mg-ATP 2, Na-GTP 0.3, and Na-Phosphocreatine 10. pH = 7.30; 278 mOsm with 0.1% biocytin.

Insular Cortex Slices: Adult rats were anesthetized by isoflurane, intracardially perfused with chilled (4℃) aCSF cutting solution and rapidly decapitated. The brain was sliced on a vibratome (VT-1000S, Leica Microsystems, Nussloch, Germany) in 300 µm coronal segments. Slices were transferred to oxygenated (95% O_2, 5% CO_2) aCSF cutting solution at 37℃ for 30 minutes followed by 30 minutes at room temperature prior to any electrophysiological recordings.

Whole-Cell Recording: Electrodes were pulled (P-1000, Sutter Instruments, CA) from 1.5 mm outer diameter borosilicate glass (Sutter Instruments, CA) and filled with recording solution. Electrode resistance was 3–5 MΩ in the bath and recordings were only included if the series resistance remained less than 30 MΩ with less than 10% change from baseline throughout the experiment. Slices were visualized using a 40x (0.75 NA) water immersion objective under infrared differential interference contrast imaging on an upright microscope (AxioExaminer D1, Zeiss, Germany). All recordings were obtained with an Axon 700B amplifier, DigiData1550, and pClamp 10 (Molecular Devices), using appropriate bridge balance and electrode-capacitance compensation. Synaptic blockers were omitted for fEPSP recordings, except where noted, and SR-95531 was excluded for

evoked inhibitory postsynaptic current (eIPSC) recordings. Insular cortex slices from adult rats were prepared as previously described (Rogers-Carter et al., 2018). Slices contained the posterior insular cortex (Bregma -1 to -2.8mm), the region of insular cortex with the greatest anatomical connectivity to the social decision making network (Rogers-Carter & Christianson, 2019). Active and passive membrane properties of deep layer insular cortex pyramidal neurons were determined in whole cell current clamp recordings. Experiments were conducted in pClamp 10 using an Axon 700B amplifier, headstage and DigiData1550 DAQ (Molecular Devices). Experimental protocols were exactly as described previously (Rogers-Carter et al., 2018). Analyses were performed using custom software written for Igor Pro (Wavemetrics Inc., Lake Oswego, OR). Active properties were quantified from single spikes by holding the neuron at -67 mV, and 2.5 ms current pulses were injected to elicit a single AP. Passive properties and input/output curves were measured by holding the membrane potential at -67 mV and injecting 1 s current pulses through the patch electrode. The amplitudes of the 1 s current injections were between -300 pA and +400 pA in 50 pA steps. All traces in which APs were elicited were used to generate input-output curves as the total number of APs per second plotted against the injected current. Resting potential was determined upon break-in. Rheobase was determined as the minimal 2.5ms current injection needed to evoke a single spike. Action potential amplitude (peak-baseline), rise rate (max mV/ms), half-width (ms), and after-depolarization (ADP, mV) were determined after subtracting the maximum sub-threshold depolarization that did not evoke an action potential. The burst ratio was computed as previously described ("burst current," (Metz et al., 2005)). in which a larger ratio indicates more current required of a transient depolarization applied during the ADP to evoke an AP. To determine the slow after hyperpolarization (sAHP), 5 APs were evoked and the sAHP was computed as the duration of hyperpolarization relative to resting potential times the peak hyperpolarization (mV x ms). Passive properties included resting potential, input resistance, sag ratio, membrane time constant, & rectification ratio. A complete list of parameters is provided in Supplementary Table 1.

<u>fEPSP</u>: Evoked field excitatory postsynaptic potentials (fEPSPs) were recorded on a 6 x 10 perforated multielectrode array (MCSMEA-S4-GR, Multichannel Systems) and integrated acquisition hardware (MCSUSB60) and analyzed with MC_RACK software (version 3.9). Slices were placed onto the array and affixed by perfusion through the perforated array. CRF (50 or 300 nM), CRF_1 antagonist CP-154526 (10 µM, the dose that prevented CRF effects on intrinsic properties and comparable to other studies (Hwa et al., 2019), and $GABA_A$ inhibitor SR-95531 (2 µM, a dose that eliminates spontaneous and evoked IPSCs in our preparation, J.A. Varela & J.P. Christianson, unpublished data) were dissolved in water or DMSO and then diluted to their final concentration in aCSF and bath applied to slices from above at 37℃. A stimulating electrode was selected in the posterior insular cortex and fEPSPs were recorded in the adjacent electrodes exhibiting robust fiber volley and linear stimulus/response properties exactly as previously (Rogers-Carter et al., 2018). Input/output curves were established at baseline (aCSF) and again after 10 min of drug application. Stimulations ranged from 0-5 V and occurred in biphasic (220 µs) 500 mV increments. Each step in the I/O curve was repeated three times for a total of 33

stimulations in each of the three conditions, and each curve was repeated 3 times at each stage of the experiment (e.g. baseline and drug).
A stimulating electrode was selected in the posterior insular cortex and fEPSPs were recorded in the adjacent electrodes exhibiting robust fiber volley and linear stimulus/response properties. Input/output curves were established at baseline (aCSF) and again after 10 min of drug application. Stimulations ranged from 0-5 V and occurred in biphasic (220 µs) 500 mV increments. Each step in the I/O curve was repeated three times for a total of 33 stimulations in each of the three conditions, and each curve was repeated 3 times at each stage of the experiment (e.g. baseline and drug). To allow comparisons between slices, fEPSP input/output curves were generated for each recording channel relative to the average fEPSP amplitude recorded at 5V at baseline. Typically, 5-10 channels were recorded on each slice which were then averaged by subject and used as replicates for between-group and treatment analysis with ANOVAs These methods are exactly as described previously.

Evoked Inhibitory Postsynaptic Currents: AMPA & NMDA receptors were blocked with 10 µm CNQX and 50 µm APV and action potentials were blocked intracellularly with the sodium channel blocker QX-314 (10mM) in the internal solution. Trains of 20 stimuli were applied at 5Hz to evoke inward eIPSCs were recorded from holding potential of -90mV as previously reported (Varela et al., 1999). Charge (area of IPSC and IPSC slope were also computed, normalized and analyzed the same way, the effect of CRF was the same with each of these approaches (data not shown).

Surgery and Behavior

Cannulation: Rats were implanted with bilateral guide cannula in the posterior insula (26-gauge, Plastics One, Roanoke VA) in the posterior insular cortex (from bregma: AP: -1.8 mm, M/L: ± 6.5 mm, D/V: -6.8 mm from skull surface) that were affixed with stainless steel screws and acrylic dental cement under isoflurane anesthesia (2-5% v/v in O_2). Immediately following surgery, rats were injected with analgesic meloxicam (1 mg/kg, Eloxiject, Henry Schein), antibiotic penicillin (12,000 units, Combi-pen 48, Covetrus) and Ringer's solution (10 mL, Covetrus). Rats were allowed 7-14 days for recovery prior to behavioral testing. Injections were 0.5 µL/side for all drugs and were infused at a rate of 1.0 µL/minute with an additional one minute diffusion time after injection. After behavioral testing concluded rats were overdosed with tribromoethanol (Sigma) and brains were immediately dissected and fresh frozen on dry ice. Coronal sections (40 µm) were stained with cresyl-violet. Rats were excluded from all analyses if their cannula placements were found to be outside the insula or if cannulas were occluded prior to injection.

Social Exploration: Each test rat was assigned to a separate plastic cage (18 cm x 24cm x 18 cm) with shaved wooden bedding and a wire lid. Rats were placed in the testing cage and allowed 1 h to acclimate. Drug injections were made 30-40 minutes prior to testing. The test began with the introduction of either a juvenile (28 ± 2 days old) or adult (50 ± 2 days old) same-sex conspecific.

Rats were then allowed to interact for five minutes and interactions were scored for social behaviors (sniffing, pinning and allogrooming) initiated by the test rat by an observer blind to treatment.

SAP test: The SAP test occurs over 4 days that all involve placement of the test rat into an empty clear plastic cage (50 x 40 x 20 cm, L x W x H) for 1h. On day 1, the rat is returned to the homecage. Day 2 concludes with a 5 min exposure to 2 naive juvenile or adult conspecifics. Conspecifics are placed into opposite ends of the testing cage in clear plastic cages (18 x 21 x 10cm, L x H x D) with one side made up of horizontal bars spaced 1 cm apart to facilitate interactions. The test rat was allowed to interact with the conspecifics for 5 min and time spent nose-nose or nose-body sniffing and reaching for the conspecific through the bars is recorded. On days 3 and 4, microinjections are made 30-40 minutes prior to tests in which one of the conspecifics placed into the cage is stressed via two 5-second 1 mA footshocks in 60 seconds immediately before testing and the other conspecific is naive to stress.

Fear Conditioning: Fear conditioning occurred in rectangular chambers made of black plastic with wire mesh lids and a floor of stainless bars attached to a shocking grid (Model H10-11R-TC-SF, Coulbourn Instruments, Whitehall, PA) housed inside a light and sound-attenuated chamber. Ventilation and masking noise of ~55dB was provided by a fan. Conditioned stimuli were delivered via a white LED array (Model LPL620WTHD, Hampton Bay) and a speaker mounted at the top of the chamber. The chamber was illuminated with 2 infrared LEDs arrays (CMVision Model IR30) mounted to the ceiling of the enclosure and overhead cameras (Model VX-5000, Microsoft, Redmond, VA) with the infrared blocking filters replaced with infrared passing filters were used to record behavior. Freezing was detected using ANY-Maze software (version 4.99, Stoelting, Wood Dale, IL) with the manufacturer's recommended settings. Apparatus and freezing analysis were described in detail previously (Chen et al., 2016; Foilb et al., 2016).

To determine if insular infusions of CRF influence fear learning, rats received bilateral cannula in the insular cortex as described above and 14 days later received either saline or CRF (0.5uL per side of 300nM CRF, as above for social interaction) 30-40 minutes before a conditioning session which consisted of a 5 minute context pre exposure followed by 4 Tone-Shock (CS-US) pairings at 1 min intervals. Tones (1kHz, 90dB) were 30s and co-terminated with a 2s footshock (1mA, scrambled). All conditioning occured in a brightly lit context. After conditioning rats were returned to the homecage. Fear recall was tested 7 days later, at a time when auditory fear conditioning using similar parameters is dependent upon the insular cortex (Foilb et al., 2016). Rats returned to the conditioning apparatus but were tested in the dark (imaging for freezing detection was conducted in infrared). Freezing was assessed in a 5 minute baseline period and then upon 20 presentations of the tone CS (30 s each, 30 s intertrial interval). To determine if insular CRF influenced fear recall, rats were prepared as above and exposed to fear conditioning drug free. 7 days after conditioning

rats received either CRF or saline infusions, 30-40 min prior to fear recall testing with 20 tone presentations as above. Rats were tested again on the 8th day without injections with the exact same testing parameters. Data are expressed as percent of time freezing over blocks of 4 CS trials.

mRNA analysis

qPCR: Punches were collected on a freezing cryostat by slicing fresh frozen brains to a coronal plane equal to Bregma -1.5mm and depressing a 1mm brain punch tool (Stoelting) through the insula such that the base of the punch intersected the rhinal fissure. The resulting punches contained the extent of insula targeted for electrophysiology, microninjection and RNAscope analysis. Punches were kept on dry ice then stored at -80℃. Total RNA was isolated using the RNAqueous-4PCR Total RNA Isolation Kit (Applied Biosystems, Foster City, CA, United States) and cDNA was synthesized using the High Capacity cDNA Reverse Transcriptase Kit (Applied Biosystems, Foster City, CA, United States). cDNA concentration was quantified using Nanodrop 2000 Spectrophotometer (Thermo Fisher Scientific), and samples were diluted accordingly. Amplification was performed using TaqMan Gene Expression Assays using gene-specific primers purchased from Thermo Fisher Scientific for *Crhr1* (Rn.10499), *Cnr1* (Rn.89774), *Crhr2* (Rn.10023), and *Crh* (Rn.10349). Reactions were performed in triplicate on a StepOnePlus Real-Time PCR System (Applied Biosystems) using standard cycling conditions, as recommended by the manufacturer. 4µl of diluted cDNA was placed in a 20µl reaction plate containing 16µl of master mix and 1x dilution of each primer. Reactions were performed with an initial holding stage of 50°C for 2 min and 95°C for 10 min, followed by 40 subsequent cycles of 15s at 95°C and 1 min at 60°C. The relative standard curve analysis method was used, with the threshold cycle (CT) (number of cycles required to reach detection threshold) determined for each reaction and the $2^{-\Delta\Delta C(t)}$ method used to determine gene expression relative to the house-keeping control gene, β-actin (Thermo Fisher Scientific, Rn.94978). Data are expressed as mean fold expression relative to females.

RNAScope: Coronal sections containing the posterior insular cortex (Bregma -1.8) were collected on a freezing cryostat at 20µm thick and mounted to SuperFrost+ slides and stored at -80℃ until processing. Probes were amplified and visualized with AMP1, AMP2, AMP3, AMP4, and DAPI, coverslipped with aqueous antifade media (Prolong Gold) and imaged on a Zeiss AxioImager Z2 microscope with a digital CCD camera (ORCA 3, Hamamatsu) using an Apotome2, 20x objective (N.A. = 0.8) and fluorescent filter cubes for DAPI (365 nm excitation, Zeiss filter 49), GFP (470/40 nm excitation, Zeiss filter 38 HE), DSRed (545/25 nm excitation, Zeiss filter 43 HE) and Cy5 (640/30 nm excitation, Zeiss filter 50). All image acquisition parameters (exposure, camera gain, and display curves were consistent for all samples. A series of multiplex, tiled mosaic images consisting of 9 z-series images per channel were stitched, deconvolved and maximum projections were saved for analysis in ImageJ. All channels were converted to binary and DAPI cells and vglut1, CRF_1 and CB_1 grains were detected using the particle counter tool. Trained observers counted the number of DAPI nuclei that were colocalized (a minimum of 3 overlapping, or adjacent grains) with each of the

targets. The left and right hemisphere of the posterior insular cortex were imaged and counted separately for each subject. To control for differences in overall cell density from section to section, the relative distribution of cell types for each subject was determined by dividing the number of nuclei expressing the target mRNA (e.g. DAPI and CRF_1) divided by the total number of cells (either DAPI or vglut1) in the region of interest.

Statistics

Sample sizes were determined based on previous work for electrophysiological tests and based on previous work and power analyses for behavioral tests. Animals were randomly assigned to experiments and within subjects designs were used throughout. To control for potential test-order effects in repeated measure experiments, subjects were randomly counterbalanced (for 2 treatment tests) or ordered in a latin square design (for 4 treatment tests). To compare differences between mean scores of social interaction and electrophysiological endpoints we used t-tests and analysis of variance (ANOVA). Individual replicate data are provided in the figures. Data were checked for normality and transformed where appropriate via log transformation, final sample sizes are indicated in the Figure Legends. In most experiments, there were within-subjects variables, which were treated as such in the analysis (paired samples t-test or repeated measures ANOVA). Main effects and interactions were deemed significant when $p < 0.05$ and all reported post hoc test *p* values are Tukey or Sidak-adjusted, to maintain an experiment-wise risk of type I errors at a = 0.05.

Supplementary Results

Insular CRF did not alter fear learning or conditioned freezing.

As both CRF and insular cortex are implicated in a range of behaviors and cognitive processes we sought to explore the generality of CRF effects in a non-social setting using Pavlovian fear conditioning (Supplemental Fig. 3). Auditory fear conditioning and later fear expression are augmented by stress and CRF (Radulovic et al., 1999) and dependent on the insular cortex when tested at temporally remote timepoints, specifically 7 days after conditioning (Foilb et al., 2016). Because CRF augmented insular excitability and mediated social interaction with stressed conspecifics, we hypothesized that CRF infusions to insular cortex during fear conditioning or fear recall would increase the strength of the learned fear response, or interfere with fear extinction, respectively. However, CRF had no effect on either fear conditioning or fear recall 7 days after acquiring fear conditioning indicating that while insular CRF is important to social behavior it does not affect fear learning and recall.

Supplementary Figures

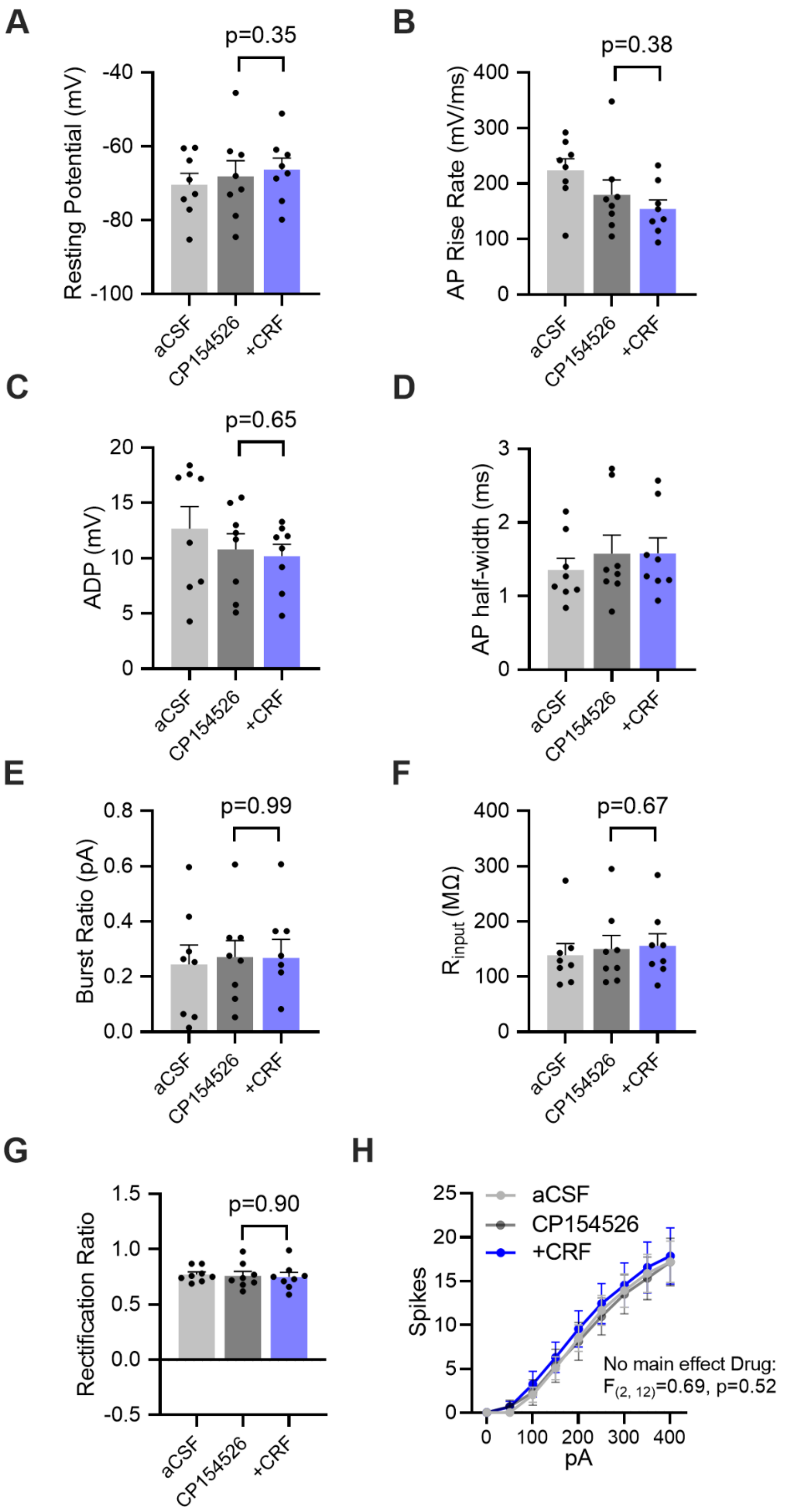

Supplemental Figure 1| CRF does not alter intrinsic membrane properties in the presence of CRF_1 receptor antagonist. Whole cell recordings were made in 8 male insular pyramidal neurons and intrinsic properties were determined exactly as in Figure 1 in regular aCSF, aCSF + CP154529 (10uM) and aCSF + CP154529 + CRF (50nM). The CRF_1 antagonist prevented changes previously observed after CRF application (see Figure 1) including **A.** resting potential **B.** action potential rise rate **C.** after depolarization **D.** action potential half-width **E.** burst ratio **F.** input resistance, **G.** rectification ratio and **H.** firing rate. All measures were evaluated for drug effects with repeated measures ANOVA. There were no significant main effects of drug and no significant posthoc comparisons between CP154526 alone and CP154526 + CRF. P values are Sidak corrected.

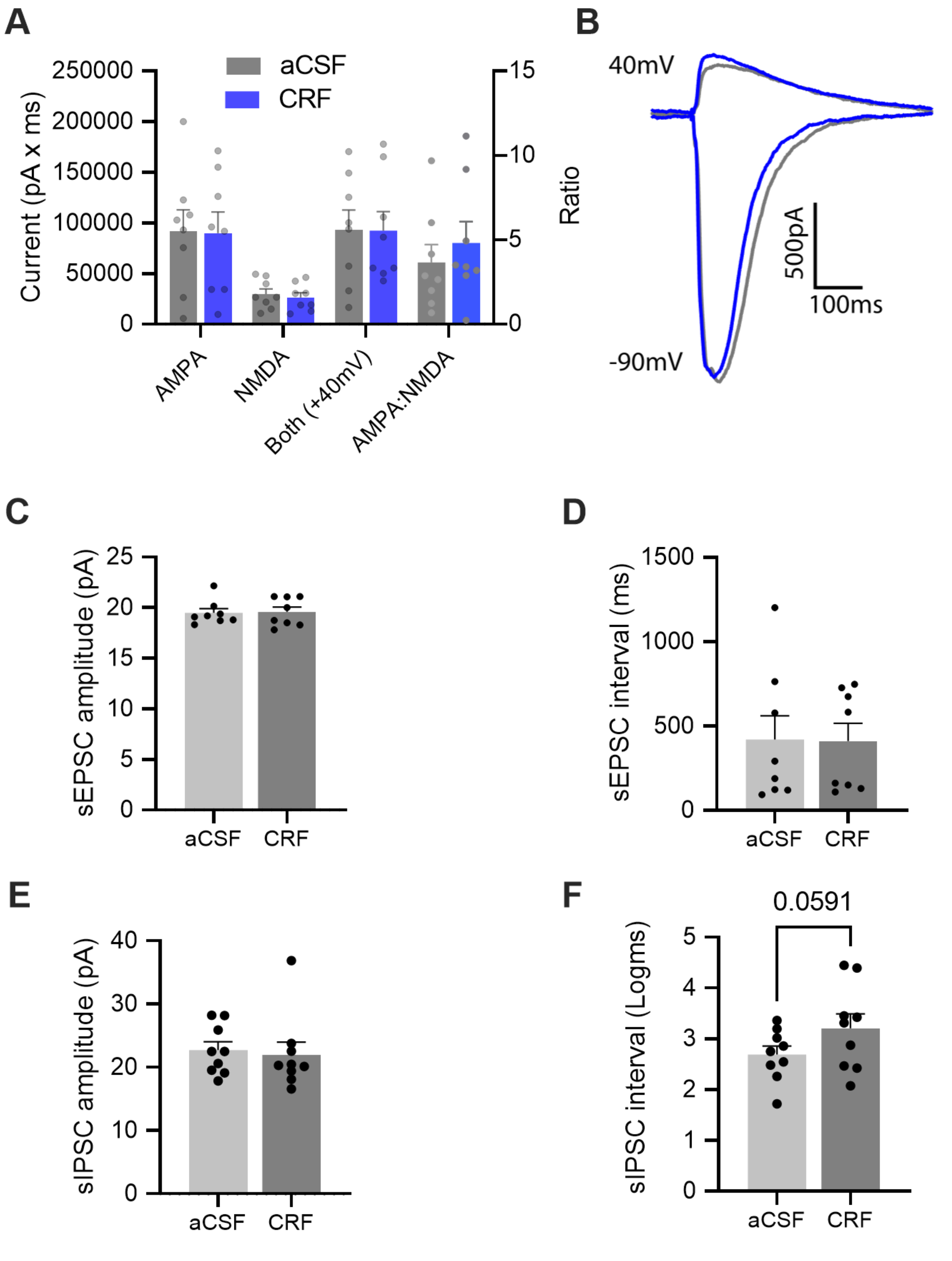

Supplemental Figure 2| CRF did not alter glutamatergic transmission. Whole cell recordings were obtained in voltage-clamp configuration in the presence of the $GABA_A$ antagonist SR-95531(2μM). Evoked excitatory postsynaptic currents (eEPSCs) were recorded at -90mV or +40mV to determine the AMPA and

AMPA+NMDA mediated components. Currents are expressed as the area under the curve (pA x ms). NMDA was determined by only considering the remaining area at +40 mV after the AMPA response at -90 mV is complete. **A.** In 8 recordings, application of CRF (50nM) did not alter AMPA or NMDA mediated currents (plotted on the left vertical-axis) or the AMPA:NMDA ratio (plotted on the right vertical-axis). **B.** Representative eEPSCs; aCSF (grey) and CRF (blue). Spontaneous excitatory postsynaptic currents (sEPSCs) were recorded in voltage-clamp recordings of 8 neurons in aCSF and then CRF. CRF did not alter the amplitude (**C**) or frequency (**D**) of sEPSCs. In a separate set of 9 neurons, spontaneous inhibitory currents (sIPSCs) were recorded in the presence of CNQX and AP5 to block glutamatergic transmission. CRF did not alter sIPSC amplitude (**E**) but did tend to increase the inter-event-interval ($p = 0.0591$, paired samples t-test after data were normalized with a Log transform).

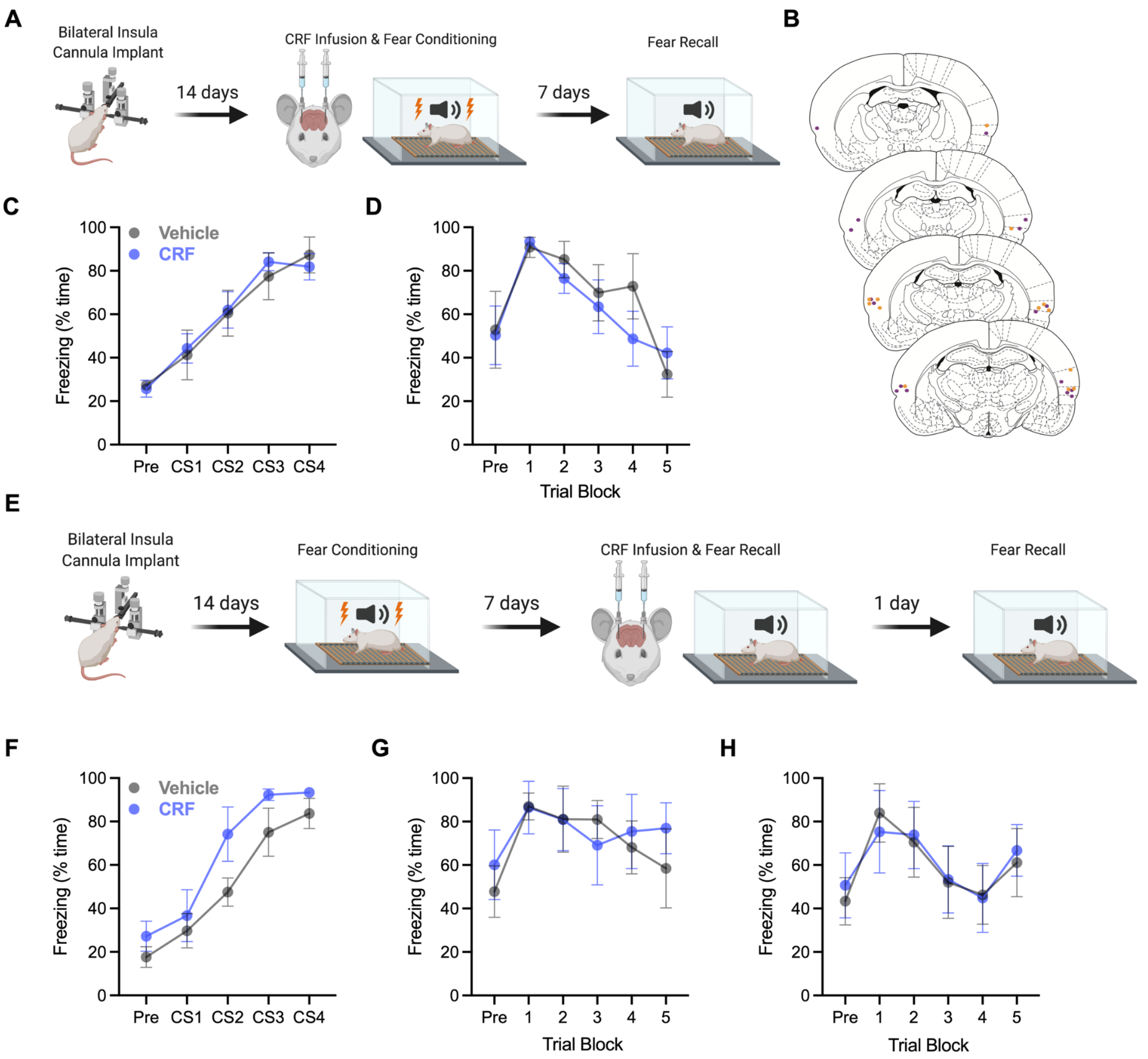

Supplemental Figure 3| CRF in the insula does not affect fear learning or recall. A. Timeline of experiment for insular CRF on fear learning and recall. Male rats were bilaterally cannulated 14 days prior to undergoing fear learning by pairing a tone with a foot shock over 4 trials. 7 days after fear learning, fear recall was tested by measuring freezing in response to the shock-paired tone. **B.** Representative locations of cannula injection sites for rats included in fear learning experiments (purple) or fear recall experiments (organe). **C.** Mean (+/- SEM) time spent freezing during the 5 min preconditioning baseline and each of the 1 minute conditioned stimulus (CS) tone-shock pairings. Fear expression was not affected by microinjection of CRF (n = 6) or saline (n = 5) 40 minutes before conditioning. **D.** Mean (+/- SEM) time spent freezing during the 5 min baseline and over blocks of 4 tone presentations. Later fear recall was not affected by CRF microinjection prior to fear learning. **E.** Timeline of experiment to determine if CRF microinjection prior to fear recall affected freezing. Male rats were implanted with bilateral insula cannula and underwent fear learning 14 days later as in A. 7 days after fear learning rats were microinjected with CRF and tested for fear recall. Rats were then retested for fear recall one day later. **F.** Mean (+/- SEM) time spent freezing during the 5 min preconditioning baseline and each of the 1 minute conditioned stimulus (CS) tone-shock pairings. Vehicle (n

= 5) and CRF (n = 5) rats showed no difference in freezing expression during conditioning as in B. **G-H.** Mean (+/- SEM) time spent freezing during the 5 min baseline and over blocks of 4 tone presentations 30 min after CRF or saline injection. Freezing was equal between saline and CRF treated rats. Freezing was equal in the recall test given 24h later. Illustrations created with BioRender.com.

Supplemental References

Chen, V. M., Foilb, A. R., & Christianson, J. P. (2016). Inactivation of ventral hippocampus interfered with cued-fear acquisition but did not influence later recall or discrimination. *Behavioural Brain Research*, *296*, 249–253. https://doi.org/10.1016/j.bbr.2015.09.008

Foilb, A. R., Flyer-Adams, J. G., Maier, S. F., & Christianson, J. P. (2016). Posterior insular cortex is necessary for conditioned inhibition of fear. *Neurobiology of Learning and Memory*, *134 Pt B*, 317–327. https://doi.org/10.1016/j.nlm.2016.08.004

Hwa, L. S., Neira, S., Pina, M. M., Pati, D., Calloway, R., & Kash, T. L. (2019). Predator odor increases avoidance and glutamatergic synaptic transmission in the prelimbic cortex via corticotropin-releasing factor receptor 1 signaling. *Neuropsychopharmacology*, *44*(4), 766–775. https://doi.org/10.1038/s41386-018-0279-2

Metz, A. E., Jarsky, T., Martina, M., & Spruston, N. (2005). R-Type Calcium Channels Contribute to Afterdepolarization and Bursting in Hippocampal CA1 Pyramidal Neurons. *Journal of Neuroscience*, *25*(24), 5763–5773. https://doi.org/10.1523/JNEUROSCI.0624-05.2005

Radulovic, J., Rühmann, A., Liepold, T., & Spiess, J. (1999). Modulation of Learning and Anxiety by Corticotropin-Releasing Factor (CRF) and Stress: Differential Roles of CRF Receptors 1 and 2. *Journal of Neuroscience*, *19*(12), 5016–5025.

Rogers-Carter, M. M., & Christianson, J. P. (2019). An insular view of the social decision-making network. *Neuroscience and Biobehavioral Reviews*, *103*, 119–132. https://doi.org/10.1016/j.neubiorev.2019.06.005

Rogers-Carter, M. M., Varela, J. A., Gribbons, K. B., Pierce, A. F., McGoey, M. T., Ritchey, M., & Christianson, J. P. (2018). Insular cortex mediates approach and avoidance responses to social affective stimuli. *Nature Neuroscience*, *21*(3), 404–414. https://doi.org/10.1038/s41593-018-0071-y

Sidiropoulou, K., Lu, F.-M., Fowler, M. A., Xiao, R., Phillips, C., Ozkan, E. D., Zhu, M. X., White, F. J., & Cooper, D. C. (2009). Dopamine modulates an mGluR5-mediated depolarization underlying prefrontal persistent activity. *Nature Neuroscience*, *12*(2), 190–199. https://doi.org/10.1038/nn.2245

Varela, J. A., Song, S., Turrigiano, G. G., & Nelson, S. B. (1999). Differential Depression at Excitatory and Inhibitory Synapses in Visual Cortex. *The Journal of Neuroscience*, *19*(11), 4293–4304. https://doi.org/10.1523/JNEUROSCI.19-11-04293.1999
